# Patient-centered precision care in anaesthesia – the PC-square (PC)^2^ approach

**DOI:** 10.1097/ACO.0000000000001343

**Published:** 2024-01-22

**Authors:** Joana Berger-Estilita, Isabel Marcolino, Finn M. Radtke

**Affiliations:** a Institute of Anaesthesiology and Intensive Care, Salemspital, Hirslanden Medical Group; b Institute for Medical Education, University of Bern, Bern, Switzerland; c CINTESIS - Center for Health Technology and Services Research, Faculty of Medicine, Porto, Portugal; d Institute of Anaesthesiology and Intensive Care, Spital Limmattal, Schlieren, Switzerland; e Department of Anaesthesia and Intensive Care, Hospital of Nykøbing Falster, University of Southern Denmark, Odense, Denmark

**Keywords:** EEG monitoring, patient outcomes, patient-centred care, personalized medicine, precision anaesthesia

## Abstract

**Purpose of review:**

This review navigates the landscape of precision anaesthesia, emphasising tailored and individualized approaches to anaesthetic administration. The aim is to elucidate precision medicine principles, applications, and potential advancements in anaesthesia. The review focuses on the current state, challenges, and transformative opportunities in precision anaesthesia.

**Recent findings:**

The review explores evidence supporting precision anaesthesia, drawing insights from neuroscientific fields. It probes the correlation between high-dose intraoperative opioids and increased postoperative consumption, highlighting how precision anaesthesia, especially through initiatives like Safe Brain Initiative (SBI), could address these issues. The SBI represents multidisciplinary collaboration in perioperative care. SBI fosters effective communication among surgical teams, anaesthesiologists, and other medical professionals.

**Summary:**

Precision anaesthesia tailors care to individual patients, incorporating genomic insights, personalised drug regimens, and advanced monitoring techniques. From EEG to cerebral/somatic oximetry, these methods enhance precision. Standardised reporting, patient-reported outcomes, and continuous quality improvement, alongside initiatives like SBI, contribute to improved patient outcomes. Precision anaesthesia, underpinned by collaborative programs, emerges as a promising avenue for enhancing perioperative care.

## INTRODUCTION

Anaesthesia is critical to surgical procedures, ensuring patient comfort and safety during surgery. However, administering anaesthesia is not without its challenges and potential adverse effects. In this chapter, we will explore the concept of precision medicine in anaesthesia, focusing on the importance of individualized care, anticipation of potential side effects and the need for precise monitoring. We will draw inspiration from recent research highlighting the gaps in current anaesthesia practices and the potential for improvement. 

**Box 1:**
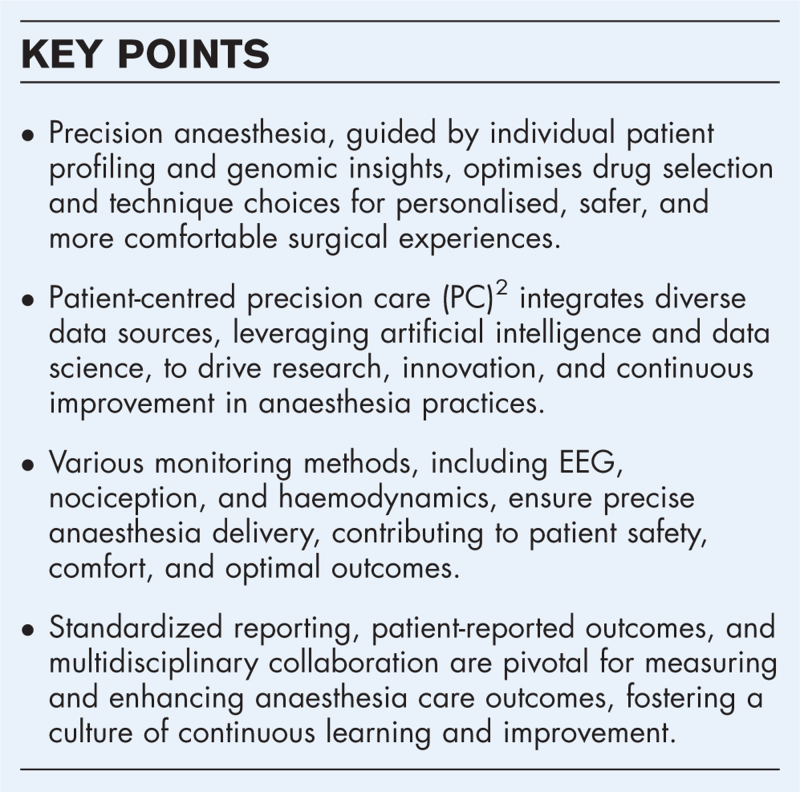
no caption available

## THE UNADDRESSED SIDE EFFECTS OF ANAESTHESIA

Anaesthesia providers often find themselves inadequately informed about the most common side effects of anaesthesia and perioperative care. These side effects include perioperative neurocognitive dysfunctions (PND) including postoperative delirium (POD), hypothermia, dehydration, and various patient-reported outcomes (PROs) such as nausea, vomiting, severe pain, stress, and anxiety [1]. Medium and long-term outcomes of anaesthesia, such as health-related quality of life, development of chronic pain, psychological/emotional and mental well being or fatigue, are not systematically reported (30). Furthermore, the impact of anaesthesia on long-term surgical outcomes or disease recurrence, or cancer recurrence has not been widely studied yet [53]. These various side effects significantly impact patient postoperative health trajectory and can potentially be mitigated or prevented [2].

Anaesthesia service providers typically do not receive structured feedback on their patients’ short-, intermediate-, and long-term outcomes, which can impede the development of a clear and holistic understanding of the cause and effect of quality care tailored to each patient's specific needs and requirements. Addressing this gap in oversight could lead to improved patient care and a more comprehensive assessment of the quality of anaesthesia provided, ultimately resulting in a positive impact on patient safety and outcomes [3^▪^].

## CHALLENGES IN IMPLEMENTING BEST PRACTICES

Despite the presence of evidence-based guidelines and best practice recommendations, implementation of these recommendations in perioperative care can be challenging [4]. Barriers include the slow adoption of guidelines, limited follow-up, and insufficient adherence monitoring [5]. Addressing these challenges requires a commitment to continuous improvements over time. Notably, patient safety and best practice guidelines are now slowly being adopted in national societies, highlighting the need for more widespread and consistent acceptance of these guidelines to enhance the quality of anaesthesia care and patient safety [6].

Patient-centred Precision Care (PC)^2^, also known as personalized or individualized medicine, is an innovative approach to medical treatment and healthcare that tailors medical decisions, practices, interventions, and therapies to the individual characteristics of each patient [7]. Instead of “one-size-fits-all” approaches, precision medicine considers the unique genetic, environmental, and lifestyle factors that influence a person's health and response to specific interventions [8].

## KEY COMPONENTS AND PRINCIPLES OF PRECISION MEDICINE

The ultimate goal of precision medicine is to improve patient outcomes, enhance the effectiveness of treatments, reduce adverse effects, and make healthcare more tailored [7]. Precision medicine allows for a more comprehensive understanding of each patient's unique genetic and clinical characteristics, paving the way for personalized treatment plans [8]. With this patient-specific data, healthcare providers can design more effective treatments less likely to cause harm, thereby improving patient outcomes [2].

## CRISPR: THE LA(TE)ST FRONTIER

Leveraging genomic information to provide highly individualized treatment plans is a revolution in healthcare. By scrutinizing an individual's DNA, healthcare providers can identify genetic variations affecting disease risk, treatment efficacy, and susceptibility to medication side effects.

The healthcare landscape has undergone a revolutionary shift with the advent of CRISPR (Clustered Regularly Interspaced Short Palindromic Repeats) technology, particularly the groundbreaking CRISPR/Cas9 system [9]. This innovation has emerged as one of gene technology's sharpest tools, akin to molecular scissors that allow precise modifications to the genetic code [10].

Traditional genetic testing methods often grappled with limitations in precision and specificity. CRISPR, however, has elevated the accuracy of genetic testing by enabling researchers and healthcare providers to pinpoint and modify specific genes or regions of the genome with unparalleled accuracy [11]. This precision is instrumental in identifying subtle genetic variations that influence disease risk, treatment efficacy, and susceptibility to medication side effects. CRISPR's efficiency in genome editing has far-reaching implications for personalized medicine. The technology's ability to make rapid and precise modifications to the genome streamlines the genetic testing process. Beyond diagnosis, CRISPR paves the way for targeted therapies based on identified genetic variations. This targeted approach enhances treatment effectiveness and contributes to cost reduction by minimizing side effects and optimizing treatment plans based on an individual's genetic makeup [12].

The NHS, for example, aims to advance genomics over the next five years by embedding it across the healthcare system [13]. The strategy emphasizes using genomics for predictive, preventive, and precision medicine, integrating genomic data into healthcare practices, and supporting scientific progress through research and innovation, aiming to empower individuals and improve health outcomes for current and future generations.

Precision medicine also plays a pivotal role in risk assessment and prevention. It can identify individuals at higher risk of certain medical conditions based on their genetic predisposition, allowing proactive measures like lifestyle changes and early screenings to detect diseases at earlier, more treatable stages [14]. Patients are actively engaged in their healthcare decisions and empowered to make informed choices about their treatment options based on personalized information [15,16]. Moreover, pharmacogenomics, an essential aspect of precision medicine, explores how an individual's genetic makeup influences their response to medication. By predicting how a patient will metabolize and respond to specific drugs, healthcare providers can guide medication selection and dosing, further minimizing adverse effects [17].

Data integration is another crucial component of precision care, as it combines diverse data sources, including genetics, clinical records, lifestyle information, and environmental factors, to create a comprehensive patient profile [14]. With advances in computational technology, artificial intelligence (AI) has given new and promising opportunities to minimize the gap between knowledge, data and patient care [18^▪^]. This multidimensional approach ensures that all relevant factors are considered in treatment decisions.

PC^2^ benefits individual patients and drives research and innovation in genetics, genomics, data science and AI [14]. PC^2^ also involves the collaboration between healthcare providers, researchers, and technology companies, pushing the boundaries of medical science and the potential for ultimately achieving the highest level of personalized care [19].

## PATIENT-CENTRED PRECISION CARE IN ANAESTHESIA – THE PC-SQUARE APPROACH

**FIGURE 1:**
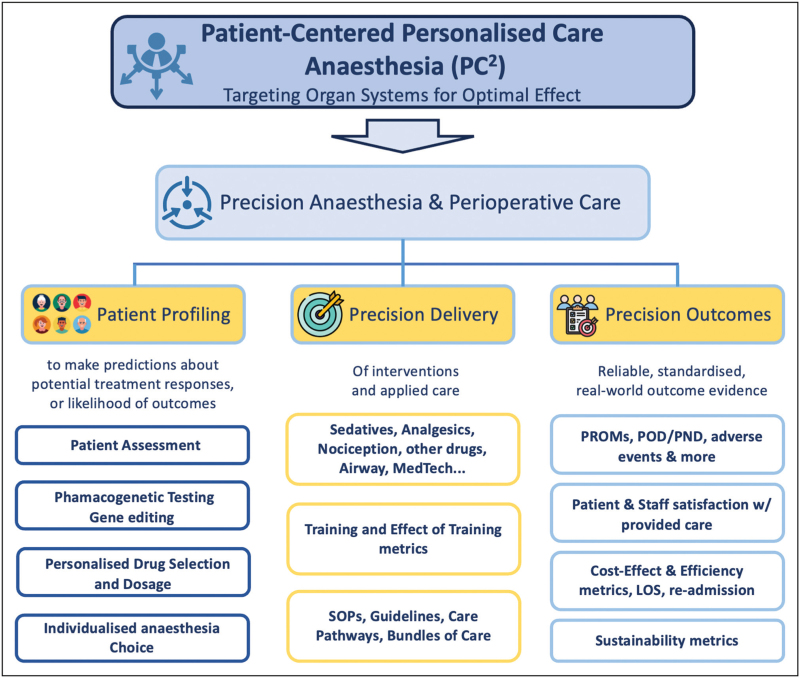
The patient-centered personalized care framework in anaesthesia (PC)^2^ diagram. The diagram focuses on organ systems for optimal effect and precision perioperative care. It integrates reliable real-world evidence and patient profiling to predict treatment responses and outcomes alongside quality metrics like patient satisfaction and cost-effectiveness. Clinical elements include the monitoring of sedation levels, personalized drug selection, and adherence to standardized procedures and guidelines.

Precision anaesthesia, like precision care in general, is a comprehensive approach to anaesthetic care that considers each patient's unique characteristics and needs. It involves tailoring anaesthesia care to individual patient's specific needs and characteristics (Fig. 1).

### Patient profiling

The process begins with a thorough patient assessment and profiling, where a detailed evaluation of the patient's medical history, current health status, genetic factors, and any specific conditions or sensitivities is conducted. Genomic information, when available, provides valuable insights into the patient's genetic predispositions and potential responses to anaesthesia and related medications. Genetic testing can identify variations that may influence a patient's metabolism of anaesthetics and other medications, aiding anaesthesia providers in selecting the most appropriate drugs and dosages. Pharmacogenomic data and pharmacokinetic and pharmacodynamic modelling guide personalised drug selection and dosage based on the patient's unique characteristics [12,20]. By individualizing drug regimens, precision anaesthesia can optimize the effectiveness of anaesthesia and minimize side effects, ensuring the best possible outcomes for each patient.

Improvements in medication management based on a patient's genotype will, for example, drive opioid usage and further advance enhanced recovery care pathways [21].

The choice of anaesthesia technique is another crucial consideration in precision anaesthesia. Whether it's general anaesthesia, regional anaesthesia, or local anaesthesia, the approach is tailored to the patient and the specific procedure. Patient comfort, safety, and individual preferences are considered when selecting the most appropriate technique [22^▪^].

Continuous monitoring of patient vital signs, depth of anaesthesia, and other relevant parameters are integral to precision anaesthesia. Advanced monitoring techniques and feedback mechanisms ensure that each patient's anaesthesia levels and physiological parameters are optimized throughout the procedure [23^▪^].

In terms of pain management, precision anaesthesia takes into account individual pain thresholds and preferences during and after surgery. Personalised pain management plans may involve tailored doses of analgesics, regional anaesthesia techniques, and multimodal pain control strategies to optimize patient comfort and recovery [24].

Precision anaesthesia also focuses on the prevention of adverse events. It aims to reduce the incidence of postoperative complications, such as postoperative delirium or cardiac adverse events, such as perioperative myocardial infarction, by identifying and mitigating risk factors specific to each patient [23^▪^]. Proactive measures may include managing pain [25], optimizing fluid balance and other hemodynamic parameters [26], and addressing factors contributing to adverse outcomes.

Ethical considerations are central to precision anaesthesia care. This includes addressing issues related to informed consent, patient autonomy, privacy, and the responsible use of genetic and genomic information, ensuring patients’ rights and well being are respected throughout the process [27,28].

To further enhance precision anaesthesia, promoting individual anaesthesia service provider-specific feedback is essential [29], allowing healthcare professionals to receive information about patient outcomes, including patient-reported outcomes and patient-reported experiences. This feedback fosters continuous individual learning and improvement and contributes to higher staff satisfaction, as it reconnects healthcare providers to the full treatment outcome of their patients, making them active participants in the patient's care journey [23^▪^].

However, a challenge that hinders the implementation of precision care in anaesthesia is the lack of clear aims or standardization in anaesthesia care [13]. Clear aims and guidelines (such as the Anesthesia Outcomes & Aims) have to be widely established [30], emphasizing the importance of precision anaesthesia and providing a framework for healthcare institutions and anaesthesia providers to follow.

### Precision delivery

Methods for delivering precision care in anaesthesia:

(1) EEG monitoring: Electroencephalography (EEG) monitoring plays a crucial role in precision anaesthesia by providing real-time insights into a patient's brain activity during surgery. Currently, the primary focus of EEG monitoring is on avoiding excessive levels of general anaesthesia that can lead to EEG burst suppression, with the superordinated goal of reducing postoperative cognitive disorders. While specific EEG features are not directly linked to intraoperative awareness, certain observations are strongly associated with cognitive outcomes [31,32]. Notably, a robust alpha band power in the EEG positively correlates with adequate anaesthesia levels [31,33]. This alpha band power is also related to improved preoperative cognitive function and a decreased risk of postoperative delirium [32]. Furthermore, it appears to convey information about nociception, as it diminishes when a potent surgical stimulus is applied without appropriate analgesic management [34]. Conversely, the presence of burst-suppression EEG patterns signals excessive anaesthesia levels, posing a potential risk for postoperative cognitive impairments. By assessing how anaesthesia medications impact the brain, healthcare providers can implement multimodal techniques to maintain the desired level of anaesthesia depth throughout the procedure [35]. This personalized approach minimizes the risks of under-sedation or over-sedation, based on the individual's neurological responses, ensuring safety during surgery and enhancing the overall comfort of the patient.
(2) Nociception monitoring: Nociception monitoring is a valuable method for assessing nociceptive stimuli during surgery [36]. By measuring the patient's physiological response to noxious stimuli, anaesthesia providers can gain a deeper understanding of the patient's stress levels. This data allows for precise adjustment of analgesic drugs to maintain optimal pain control, ensuring patient comfort and contributing to a smoother recovery process by reducing side effects of opioids [37] and other analgetic medication.
(3) Cerebral/somatic oximetry monitoring: In procedures where maintaining adequate cerebral [38] or somatic oxygenation [39] is critical, such as beach chair [40] or robotic procedures, cerebral/somatic oximetry monitoring is employed. It measures oxygen saturation in cerebral and somatic tissues, assisting anaesthesia providers in ensuring proper oxygen supply to at-risk areas. This precision in oxygen monitoring level contributes to the patient's safety and prevention of POD [41].
(4) Hemodynamic monitoring: Monitoring haemodynamics involves assessing the patient's cardiovascular status and optimizing fluid balance. Precise control of blood pressure, volume status, cardiac function, and personalized catecholamine dosing is essential to tailor the anaesthesia to each patient's unique needs and maintain hemodynamic stability throughout the surgery [42,43]. This meticulous monitoring of cardiovascular parameters contributes to patient safety and helps prevent adverse events.
(5) Continuous SpO_2_ monitoring and capnography: Capnography is an indispensable technology for measuring respiratory function during surgery. By monitoring the patient's carbon dioxide levels in exhaled breath, anaesthesia providers gain insight into the ventilation provided and the overall respiratory status of the patient. This information and continuous SpO_2_ monitoring are crucial for ensuring the patient receives adequate oxygenation and ventilation throughout the surgical procedure, contributing to patient safety and optimal oxygen supply to vital organs.
(6) Continuous temperature monitoring: Continuous temperature monitoring enables anaesthesia providers to make real-time adjustments to prevent hypothermia or hyperthermia. Maintaining the patient's optimal body temperature is paramount for a successful surgical outcome and for the patient's well being and safety [44].
(7) Noise monitoring: Minimizing disturbances during surgery is essential for patient comfort and safety. Noise monitoring is a component of precision anaesthesia [23^▪^], as it emphasizes efforts to maintain a calm and peaceful environment in the operating room. Reducing noise levels and distractions enhances the patient's experience and contributes to a smoother surgical process [45].

### Precision outcomes

Measurement and reporting of outcome and side-effects and quality improvement

Precise measurement and reporting of outcomes and side effects are essential for optimizing anaesthesia care within the framework of multidisciplinary approaches in perioperative care. One of such first multidisciplinary approaches was the Enhanced Recovery After Surgery (ERAS) program for the major abdominal surgery Field [46]; ERAS pathways reduce the delay until full recovery and have been shown to reduce postoperative morbidity and as a consequence, length of hospital stay and related costs. The more recently implemented Safe Brain Initiative (SBI) [23^▪^] represents an innovative, cost-effective, and patient-centred approach to perioperative care integrating PROMs and systematic feedback mechanisms to prevent postoperative delirium (POD) and neurocognitive disorders (NCD).

For these programs, several key factors have to be considered:

(1) Standardized reporting: Implementing standardised reporting methods to consistently capture and document patient outcomes and side effects. This systematic approach allows for the thorough analysis and comparison of anaesthesia care across different cases, helping identify areas for improvement [46].
(2) Patient-reported outcomes (PROs): Include patient-reported outcomes in the assessment process. Patients’ subjective experiences, such as pain levels, stress, and anxiety, are valuable metrics in assessing the quality of anaesthesia care. Standardized scales can be used to measure and report PROs, ensuring a patient-centred approach to outcome measurement [23^▪^].
(3) Data integration: Collect data from various sources, including anaesthesia records, patient-reported outcomes (PROs), and clinical data, to create a comprehensive patient profile. Integrating this data into the anaesthesia care pathway enhances understanding of the patient's condition and response to anaesthesia [47].
(4) Continuous quality improvement: Establish instruments for continuous quality improvement within the anaesthesia department. Regularly review processes to identify opportunities to refine anaesthesia care, contributing to ongoing improvement in patient outcomes and side-effect management [46].
(5) Education and training: Ensure that anaesthesia providers receive ongoing education and training in the principles and practices of precision anaesthesia. This fosters a culture of continuous learning and improvement, ultimately contributing to better patient outcomes and preventing side effects [48^▪▪^].
(6) Multidisciplinary collaboration: Collaborate with other healthcare professionals as part of a holistic treatment pathway, such as seen in the SBI program [23^▪^]. Effective communication and coordination among surgical teams, anaesthesiologists, nursing staff, and other medical professionals are essential for comprehensive patient care and outcome measurement.

### Assessing cost-effectiveness

So far, most studies concluded that the PM interventions were at least cost-effective compared to usual care [49]. However, introducing precision medicine strategies into routine practice will require robust economic evidence. Decision-makers need to understand the value of a precision medicine strategy compared to alternative treatment methods. Determining this value poses unique methodological challenges that might require new solutions which enable patient-level analyses and capture the dynamics of interventions in complex systems specific to the context of healthcare service delivery [50–52].

## CONCLUSION

Precision care in anaesthesia represents a promising approach to enhancing patient care and minimising adverse events associated with anaesthesia. By tailoring anaesthesia to individual patients and implementing precise monitoring and reporting of outcomes, healthcare providers can work towards improving clinical and long-term outcomes and optimising the patient's perioperative experience. While challenges persist, the commitment to implementing and sustaining these practices is crucial for advancing the field of anaesthesia and ensuring the well being of patients.

## Acknowledgements

*None.*

### Financial support and sponsorship

*None.*

### Conflicts of interest

*J.B.E. is an associate editor for BMC Medical Education and has received travel expenses from Medtronic for the Save the Brain Initiative training. F.R. has received speaker fees and Educational Grants for the Safe Brain Initiative from Medtronic. He belongs to the advisory board of Medtronic and GE Healthcare. I.M. declares that she has no known competing financial interests or personal relationships that could have appeared to influence the work reported in this paper.*

## REFERENCES AND RECOMMENDED READING

Papers of particular interest, published within the annual period of review, have been highlighted as:

▪ of special interest
▪▪ of outstanding interest
